# Consumers Control Diversity and Functioning of a Natural Marine Ecosystem

**DOI:** 10.1371/journal.pone.0005291

**Published:** 2009-04-22

**Authors:** Andrew H. Altieri, Geoffrey C. Trussell, Patrick J. Ewanchuk, Genevieve Bernatchez, Matthew E. S. Bracken

**Affiliations:** Marine Science Center, Northeastern University, Nahant, Massachusetts, United States of America; University of California San Diego, United States of America

## Abstract

**Background:**

Our understanding of the functional consequences of changes in biodiversity has been hampered by several limitations of previous work, including limited attention to trophic interactions, a focus on species richness rather than evenness, and the use of artificially assembled communities.

**Methodology and Principal Findings:**

In this study, we manipulated the density of an herbivorous snail in natural tide pools and allowed seaweed communities to assemble in an ecologically relevant and non-random manner. Seaweed species evenness and biomass-specific primary productivity (mg O_2_ h^−1^ g^−1^) were higher in tide pools with snails because snails preferentially consumed an otherwise dominant seaweed species that can reduce biomass-specific productivity rates of algal assemblages. Although snails reduced overall seaweed biomass in tide pools, they did not affect gross primary productivity at the scale of tide pools (mg O_2_ h^−1^ pool^−1^ or mg O_2_ h^−1^ m^−2^) because of the enhanced biomass-specific productivity associated with grazer-mediated increases in algal evenness.

**Significance:**

Our results suggest that increased attention to trophic interactions, diversity measures other than richness, and particularly the effects of consumers on evenness and primary productivity, will improve our understanding of the relationship between diversity and ecosystem functioning and allow more effective links between experimental results and real-world changes in biodiversity.

## Introduction

Biodiversity influences ecosystem functions and services (e.g., primary productivity, nutrient cycling, food production) because of species' traits and interactions in mixed assemblages [1], [2]. Our understanding of the links between biodiversity and ecosystem function has been predominantly shaped by experiments involving assembled communities where species are present at the same relative density; thus, diversity is often defined solely in terms of species richness [3]–[5]. This emphasis on richness rather than other measures of diversity, particularly in mesocosm or horticultural settings, has limited our ability to generalize experimental results to natural systems where ecological processes determine the composition and relative species abundance (evenness) of plant assemblages [5]–[10].

Mounting evidence indicates that evenness is a component of biodiversity that can influence ecosystem function [4]–[6], [10], [11]. Attention to the link between evenness and ecosystem function is critical because ecological interactions and human activities, such as targeted harvests, often modify evenness by skewing species abundances rather than by reducing species richness via extinction [5], [12], [13]. To date, our understanding of the importance of evenness effects on ecosystem function is largely based on experimental plant communities, where the relative abundance of primary producers is directly manipulated [6]. This approach has led to important insights, but like randomly constructed experiments examining richness effects [e.g.], [14,15], it does not fully incorporate ecological interactions such as herbivory that generate natural patterns of evenness and richness [7], [13]. Although consumers can mediate the abundance and species composition of primary producers [8], [16]–[19] and thereby influence productivity and other ecosystem functions, the generality of these consumer effects across natural ecosystems remains poorly understood [20]. A better understanding of biodiversity-functioning relationships requires field experiments where variation in ecological interaction strengths are allowed to drive the emergence of natural, non-random patterns of diversity [7], [8], [21]–[23].

In this study, we manipulated the abundance of a dominant herbivorous snail (*Littorina littorea*, hereafter *Littorina*) in rocky shore tide pool communities and then measured the productivity and biodiversity (richness, evenness, and diversity) of the resulting seaweed assemblages. *Littorina* grazing has long been recognized as a driver of intertidal algal diversity [19], and recent experiments have demonstrated the general importance of algal species richness and identity in mediating primary productivity [24], [25]. We removed existing algal biomass from tide pools to mimic natural winter storm disturbance and then allowed algal communities to develop in response to different snail densities. This approach resulted in ecologically realistic, non-random assemblages that reflected the trophic structure, dispersal, disturbance, and other processes of a natural system. Moreover, tide pools isolated at low tide provided a unique opportunity to measure community composition and productivity at a naturally defined spatial scale. We found that consumers had strong effects on evenness (but not richness) that were accompanied by increased rates of algal productivity. Our results highlight the importance of examining realistic, consumer-driven changes in evenness to better understand the links between biodiversity and ecosystem functioning.

## Results

**Figure 1:**
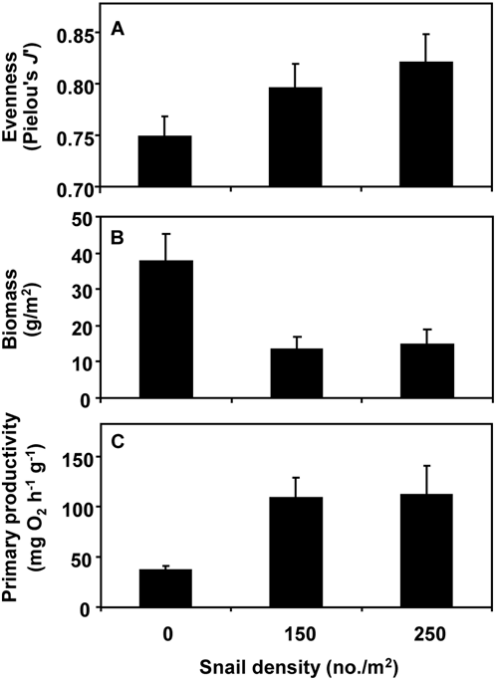
Mean (+SE) (A) species evenness, (B) final standing crop biomass, and (C) biomass-specific productivity of tide pool seaweed communities at different snail densities. Since snail enhancement of both evenness (*P* = 0.05) and biomass-specific productivity (*P* = 0.01) counteracted their reduction of algal biomass (*P*<0.0001), productivity at the scale of the entire pool did not differ among snail treatments (*P* = 0.22).

**Figure 2:**
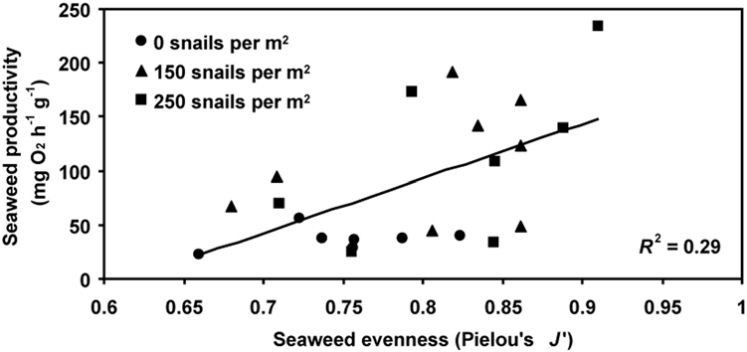
Influence of seaweed species evenness on biomass-specific productivity in tide pools. The positive relationship between biomass-specific productivity and seaweed species evenness (*P* = 0.001) held even after accounting for the potential effects of biomass variation on productivity (see *Results*). Symbols indicate pools of different snail densities: 0 per m^2^ (circles), 150 per m^2^ (triangles), and 250 per m^2^ (squares).

Grazing by snails increased tide pool seaweed species evenness (*P* = 0.05, Table S1, Fig. 1A) and biomass (g m^−2^; P<0.001, Table S1, Fig. 1B). Increases in snail density were also associated with enhanced biomass-specific productivity (mg O_2_ h^−1^ g^−1^) of tide pool macroalgae (*P* = 0.01, Table S2, Fig. 1C). This result was not due to snail respiration, because we found no relationship between snail density and respiration (O_2_ consumption) rates in tide pools (*F*_1,20_ = 0.007, *P* = 0.933). Even after accounting for the inhibiting effect of algal biomass (g/L) on biomass-specific productivity (*F*_1,19_ = 20.7, *P*<0.001), there was a positive relationship between algal species evenness and biomass-specific productivity (*F*_1,19_ = 15.0, *P* = 0.001; Fig. 2). Due to this grazer-mediated enhancement of biomass-specific productivity, snails had no effect on whole tide pool gross productivity (*P* = 0.22, Table S2) or area-specific productivity (*P* = 0.47, Table S2), despite their reduction of algal standing crop biomass.

**Figure 3:**
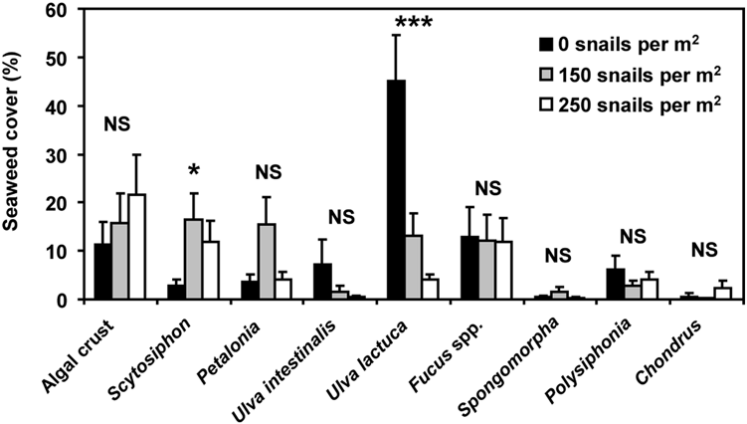
Mean (+SE) abundance of tidepool algae at different snail densities. Although high (250 per m^2^) and low (150 per m^2^) snail densities affected the abundance of specific algae differently, they generated similar patterns of species evenness and productivity. Symbols above bars indicate species-specific effects of density on cover: “***” indicates *P*<0.0001, “*” indicates *P*<0.05, and “NS” indicates that there were no differences between treatments for that seaweed species.

Snails did not influence any metrics of diversity other than evenness. Species richness (*S*), Shannon-Wiener's *H′*, and Simpson's *D* did not vary across snail treatments (*P*≥0.73 for all analyses, Table S1). Species identity was also similar across all levels of snail grazing, with 11 of 15 algal taxa found at all 3 snail densities, and the other 4 species occurring only rarely (each in 3 or fewer of the 36 experimental tide pools). Although high and low snail densities similarly increased algal species evenness, the snail density treatments had unique effects on the relative abundance of several algal species, particularly *Ulva* and *Scytosiphon* (*P*<0.001, Table S3, Fig. 3).

## Discussion

Our experiments in natural tide pools revealed that consumers increased algal species evenness and enhanced biomass-specific primary productivity. Both algal species evenness and biomass-specific productivity were higher in tide pools with herbivorous snails than pools where snails were absent (Fig. 1). The positive relationship between algal evenness and biomass-specific productivity (Fig. 2) persisted even after adjusting for the effect of snails on algal biomass.

Enhanced rates of biomass-specific productivity associated with selective snail grazing had important consequences for gross primary productivity at the scale of the entire tide pool. Snails decreased the overall standing crop biomass of macroalgae (Fig. 1B). However, both whole-pool primary productivity (mg O_2_ h^−1^ pool^−1^) and area-specific productivity (mg O_2_ h^−1^ m^−2^) were unaffected by snail density. This decoupling of productivity from algal biomass can be explained by the higher biomass-specific productivity associated with grazer mediated increases in evenness that compensated for overall reductions in seaweed biomass. Biodiversity-ecosystem function investigations commonly quantify biomass as their measure of productivity [1], [2], [26]. However, recent studies have suggested that standing crop biomass is an incomplete proxy for ecosystem functioning, particularly when experiments incorporate tropic interactions that often strongly shape natural ecosystems [27], [28]. Incorporating both short-term physiological measures of productivity and longer-term measures of standing stock, as in our study, can provide complementary insights into the mechanisms underlying the relationship between biodiversity and ecosystem function [9].

Although snails strongly influenced algal species evenness, they did not affect other aspects of diversity (i.e., species identity, species richness, Shannon-Wiener's *H′*, and Simpson's *D*). These results suggest that larger-scale processes and the regional species pool drove species composition in tide pools over the course of our 6-month experiment [29], whereas snail grazing primarily affected the evenness of algal species. Hence, consumers may mediate relationships between biodiversity and ecosystem function through their effects on an aspect of diversity (evenness) that is typically not considered in experimentally constructed communities [4], [5], [23].

In addition to their top-down effects on producer biomass, consumers can influence nutrient availability in tide pools [30], [31], and consumer-mediated nutrient inputs can affect productivity in both terrestrial and marine ecosystems [31], [32]. However, the lower evenness and biomass-specific productivity of seaweed assemblages we observed in pools without snails (Fig. 1) likely occurred because the release from grazing allowed a competitively dominant alga, *Ulva lactuca*, to occupy a larger proportion of the seaweed assemblage (Fig. 3). Despite the high biomass-specific photosynthesis rates of *Ulva* relative to other species in the laboratory under saturating flow conditions [33], the lower productivity we observed in *Ulva*-dominated tide pools is consistent with *Ulva's* interactions in the field. *Ulva* uses bicarbonate as a carbon source, which can elevate tide pool pH and reduce inorganic carbon levels, thereby causing a 5-fold reduction in the photosynthesis rates of seaweeds such as *Chondrus* and *Fucus* [34]. *Ulva* also can inhibit its own photosynthesis and that of other seaweeds because its sheet-like morphology limits light penetration below the top layer of the canopy [35]. These shading effects—which are not typically observed in the laboratory due to architectural differences between thallus pieces, whole thalli, and multi-species assemblages [36]—are likely to be even more pronounced in the still-water conditions of tide pools. *Ulva* is highly preferred by *Littorina* [19], and we observed high abundances of *Ulva* only when snails were absent. Thus, the positive effect of algal evenness on productivity may be linked to trade-offs between algal palatability and competitive ability.

Previous work has not explicitly considered the influence of variation in grazer abundance on the species evenness and productivity of primary producers, but several studies have examined aspects of this relationship. Selective grazing on algal functional groups affects the evenness of tide pool algae [37], and the presence or absence of different grazer guilds can drive variation in the dominant functional groups of seaweeds, with strong consequences for biomass-specific productivity [38]. Bruno and O'Connor [39] found that consumers affected algal evenness in a mesocosm study, but the relationship between evenness and productivity was unclear because evenness did not vary independently of richness. In contrast, Schmitz [10] found that subtle differences in evenness had large consequences for ecosystem function in terrestrial old-field communities, where grazers decoupled evenness from other diversity indices.

The links we describe between consumers, productivity, and evenness differ in several ways from the results of previous experiments in terrestrial ecosystems. We found that snails increased evenness and biomass-specific productivity by selectively grazing the highly abundant and palatable *Ulva*, which apparently suppresses the productivity of neighboring algae. In terrestrial ecosystems, insect grazing can decrease plant evenness if insects target moderately abundant species [40]. Moreover, insect-mediated increases in plant evenness can result in a negative relationship between evenness and productivity if insects selectively consume dominant, highly productive plants [10].

Recent studies suggest that the importance of producer evenness may rival the effects of richness in determining the functional consequences of biodiversity change [e.g.], [4], [6], [10,11]. We extended this perspective by considering how grazer abundance affects primary productivity and found a relationship between grazer density and seaweed evenness that had significant consequences for ecosystem productivity. Our findings highlight the importance of trophic interactions in determining diversity-functioning relationships and suggest that predicting the ecosystem-level consequences of extinctions at higher trophic levels is not likely to be a straightforward endeavor. Studies that consider the role of trophic interactions in natural food webs, rather than the ecosystem consequences of randomly assembled diversity at a single trophic level, are necessary to more fully understand the real-world consequences of changes in biodiversity.

## Materials and Methods

### Experimental design

We examined the influence of grazer (*Littorina*) abundance on seaweed diversity and productivity in tide pools on the rocky shores of Nahant, Massachusetts, USA (42.4°N, 70.9°W). Our experimental tide pools were located in the lower intertidal zone (0 to 1 m above mean lower-low water) and had an average volume of 147 (±15.3 SE) L.

Twelve experimental tide pools were randomly assigned to each of three snail density treatments: 0, 150, and 250 individuals per m^2^. These densities are known to create differences in tide pool algal diversity and are within the range commonly observed in New England tide pools [∼3 to 286 per m^2^; ref. 19]. Snail densities were established and maintained as necessary by manual removals and additions. To minimize snail immigration and emigration, we used bolts and washers to secure a 10 cm wide border of 7 mm galvanized steel mesh flush with the substratum around the rim of each pool.

In March 2004, prior to establishing our experimental snail treatments, we cleared all biomass from each tide pool with wire brushes and propane torches. Pools are often scoured clean by winter storms, so our clearing procedure simulated natural disturbance dynamics. We concluded the experiment and collected data on algal productivity and community composition in September 2004. Conducting an experiment for a single, 6-month growing season is relevant in this system because physical disturbance is frequent (especially during winter storms) and rates of growth, senescence, and compositional turnover in marine seaweed assemblages are rapid relative to terrestrial plant systems [24]. Moreover, previous work by Lubchenco [19] found that snail grazing can lead to rapid changes in tide pool algal diversity.

### Data collection

At the end of the experiment, we collected algal productivity data by conducting whole-pool incubations [41]. We calculated *gross primary productivity* by adding algal *respiration* (O_2_ consumption in the dark) and *net primary productivity* (O_2_ production in sunlight). While tide pools were isolated at low tide, we recorded the initial O_2_ concentration of the tide pool water (mg O_2_ L^−1^) using an HQ-10 meter with an LDO-probe (Hach Company, Loveland, Colorado, USA). We then covered the pools with opaque tarpaulins for a 1–2 h dark incubation. After this incubation, we recorded the O_2_ concentration again and then allowed a 1 h light incubation before taking a third O_2_ measurement. We multiplied productivity rates by the volume of each tide pool. Differences between the first and second O_2_ measurements provided an estimate of respiration rates (mg O_2_ h^−1^), and differences between the second and third measurements gave an estimate of net productivity. Gross primary productivity (mg O_2_ h^−1^ pool^−1^) was also divided by tide pool area to calculate area-specific primary productivity (mg O_2_ h^−1^ m^−2^) and by the dry seaweed biomass in each tide pool (see below) to calculate biomass-specific primary productivity (mg O_2_ h^−1^ g^−1^).

Initial measurements were made prior to sunrise to avoid O_2_ super-saturation of tide pools, and all O_2_ measurements were made on a windless day to minimize O_2_ exchange between tide pools and the atmosphere. Although the pools likely contained phytoplankton, Nielsen [41] found that phytoplankton contribute negligibly to tide pool oxygen fluxes over this time scale. It is also unlikely that *Littorina* respiration had an appreciable effect on tide pool productivity estimates for 3 reasons. First, we found no relationship between snail density (no. per m^2^) and tide pool respiration rates (see *Results*). Second, within a tidepool, the effects of *Littorina* respiration during the dark and light incubations likely cancelled out one another when summing the two terms for the gross productivity calculation because snail respiration would have increased the dark incubation and decreased the light incubation oxygen flux terms to a similar degree. Third, when comparing tidepools with and without *Littorina*, snail respiration, which can increase slightly just after sunrise (the time of our incubations) [42], would have marginally reduced net productivity rates, making our estimates of consumer enhancement of biomass-specific algal productivity conservative. Oxygen measurements that were compromised by the incoming tide were excluded from productivity analyses, leaving sample sizes of 7, 8, and 7 pools for the 0, 150, and 250 snails per m^2^ treatments, respectively.

Algal diversity was estimated by a point intercept method. One day after the productivity measurements, we randomly placed three 25×25 cm quadrats with 25 points in each tide pool. We recorded the number of points in each quadrat that fell over a given species of algae. All species of fleshy macroalgae were identified to the species level except *Fucus* spp., which were identified to the genus level, and non-upright forms, which were grouped into larger taxonomic categories such as diatoms and coralline algae due to logistical constraints of field sampling within one tide series. The algae within the three randomly placed quadrats were then scraped from the rock, dried to constant mass, and weighed. The biomass of algae in the quadrats was scaled to tide pool area to estimate the total algal biomass of each pool.

### Data analyses

We calculated several metrics of diversity for each tide pool. *Richness* (*S*) was the total number of algal species observed within the point intercept quadrats of each tide pool. *Diversity* (which combines richness and evenness) was calculated in two different ways: Shannon-Wiener's *H′* = −∑*p*_i_log_2_ *p*_i_ and Simpson's *D* = 1/∑*p*_i_^2^, where *p* is the proportional abundance of a given species in each plot. *Evenness* was calculated from actual and maximum *H′* values as Pielou's *J′* = (−∑*p*_i_log_2_ *p*_i_)/log_2_ *S*, where *S* is species richness. Evenness values for samples with 0 or 1 species were undefined and were not included in analyses [43].

Data were analyzed with R statistical software v2.8.1 (R Foundation for Statistical Computing, Vienna, Austria). The effects of snail density on algal diversity (*S*, *H′*, *D*, and *J′*) and algal biomass, productivity rates, and relative abundance of algal species were analyzed with permutational MANOVA (PERMANOVA) [44], with snail density as a fixed factor. Post-hoc analyses of nested response variables (algal diversity, biomass, and species abundance) were conducted with linear mixed effects models, and post-hoc analyses of pool-wide response variables (productivity) were conducted with ANOVA. Residual plots were visually inspected and data were transformed when necessary to meet the assumptions of statistical tests [45]. To minimize sampling error due to rare algal species, which occurred in very low abundances in a few tide pools, only the algal species that occurred in 8 or more of the experimental tide pools (9 spp.) were included in the analysis of algal species abundance.

## Supporting Information

Table S1 Effects of snail density on algal biomass and measures of biodiversity, including richness, diversity, and evenness.
(0.06 MB DOC)

Table S2 Effects of snail density on algal productivity.
(0.12 MB DOC)

Table S3 Effects of snail density on algal species abundances.
(0.09 MB DOC)
