# Supplementary material for: Consumers Control Diversity and Functioning of a Natural Marine Ecosystem
Source: PLoS One. 2009 Apr 22;4(4):e5291. doi: 10.1371/journal.pone.0005291 (PMC2668074; doi:10.1371/journal.pone.0005291)
Supplement: Table S1 — Effects of snail density on algal biomass and measures of biodiversity, including richness, diversity, and evenness. (0.06 MB DOC) [file pone.0005291.s001.doc]

**Supplementary Table S1**

Effects of snail density on algal biomass and measures of biodiversity, including richness, diversity, and evenness.

Table S1a. Statistical summary of PERMANOVA of snail density effects on algal biomass and measures of biodiversity.

| Source | DF | MS | *F* | *P* |
| --- | --- | --- | --- | --- |
| Snail density | 1 | 0.2590 | 17.77 | <0.0001 |
| Pool (snail density) | 31 | 0.0594 | 4.076 | <0.0001 |
| Error | 63 | 0.0146 |  |  |

Table S1b. Statistical summary of post-hoc analysis (linear mixed effects model) of snail density effects on algal biomass and measures of biodiversity.

| Source | DF | MS | VarComp | L. ratio | *P* |
| --- | --- | --- | --- | --- | --- |
| Biomass |  |  |  |  |  |
| Snail density | 1 | 0.0010 | — | 12.50 | < 0.001 |
| Pool (snail density) | 1 | — | 0.02 | 58.34 | < 0.0001 |
| Error | — | — | 0.01 | — | — |
| Richness (*S*) |  |  |  |  |  |
| Snail density | 1 | 7.85E-5 | — | 0.12 | 0.73 |
| Pool (snail density) | 1 | — | 0.01 | 58.34 | < 0.0001 |
| Error | — | — | 0.01 | — | — |
| Shannon-Wiener’s *H*’ |  |  |  |  |  |
| Snail density | 1 | 3.09E-5 | — | 0.03 | 0.86 |
| Pool (snail density) | 1 | — | 0.01 | 26.03 | < 0.0001 |
| Error | — | — | 0.01 | — | — |
| Simpson’s *D* |  |  |  |  |  |
| Snail density | 1 | 5.37E-6 | — | 0.00 | 0.99 |
| Pool (snail density) | 1 | — | 0.03 | 26.97 | < 0.0001 |
| Error | — | — | 0.02 | — | — |
| Pielou’s *J*’ |  |  |  |  |  |
| Snail density | 1 | 0.0003 | — | — | 0.05 |
| Pool (snail density) | 1 | — | 0.01 | 3.84 | < 0.01 |
| Error | — | — | 0.01 | 8.16 | — |
