# Supplementary material for: Consumers Control Diversity and Functioning of a Natural Marine Ecosystem
Source: PLoS One. 2009 Apr 22;4(4):e5291. doi: 10.1371/journal.pone.0005291 (PMC2668074; doi:10.1371/journal.pone.0005291)
Supplement: Table S2 — Effects of snail density on algal productivity. (0.12 MB DOC) [file pone.0005291.s002.doc]

**Supplementary Table S2**

Effects of snail density on algal productivity.

Table S2a. Statistical summary of PERMANOVA of snail density effects on algal productivity.

| Source | DF | MS | *F* | *P* |
| --- | --- | --- | --- | --- |
| Snail density | 1 | 221945.18 | 1944.03 | < 0.0001 |
| Error | 20 | 111.31 |  |  |

Table S2b. Statistical summary of post-hoc analysis (ANOVA) of snail density effects on measures of algal productivity.

| Source | DF | MS | *F* | *P* |
| --- | --- | --- | --- | --- |
| Biomass-specific productivity |  |  |  |  |
| Snail density | 1 | 0.1814 | 8.05 | 0.01 |
| Error | 20 | 0.0225 |  |  |
| Tide pool gross productivity |  |  |  |  |
| Snail density | 1 | 0.0005 | 1.59 | 0.22 |
| Error | 20 | 0.0003 |  |  |
| Area-specific productivity |  |  |  |  |
| Snail density | 1 | 0.0206 | 0.53 | 0.47 |
| Error | 20 | 0.0388 |  |  |

Table S3a. Statistical summary of PERMANOVA of snail density effects on algal species abundances.

| Source | DF | MS | *F* | *P* |
| --- | --- | --- | --- | --- |
| Snail density | 1 | 6.8103 | 47.70 | <0.0001 |
| Pool (snail density) | 32 | 1.2504 | 8.76 | <0.0001 |
| Error | 68 | 0.1428 |  |  |

Table S3b. Statistical summary of post-hoc analysis (linear mixed effects model) of snail density effects on abundance of each algal species.

| Source | DF | MS | VarComp | L. ratio | *P* |
| --- | --- | --- | --- | --- | --- |
| Algal crusts |  |  |  |  |  |
| Snail density | 1 | 0.0005 | — | 0.91 | 0.34 |
| Pool (snail density) | 1 | — | 0.08 | 63.66 | < 0.0001 |
| Error | — | — | 0.03 | — | — |
|  |  |  |  |  |  |
| *Scytosiphon* |  |  |  |  |  |
| Snail density | 1 | 0.0007 | — | 3.97 | < 0.05 |
| Pool (snail density) | 1 | — | 0.04 | 37.81 | < 0.0001 |
| Error | — | — | 0.03 | — | — |
|  |  |  |  |  |  |
| *Petalonia* |  |  |  |  |  |
| Snail density | 1 | 0.0002 | — | 0.21 | 0.65 |
| Pool (snail density) | 1 | — | 0.05 | 64.01 | < 0.0001 |
| Error | — | — | 0.02 | — | — |
|  |  |  |  |  |  |
| *Ulva intestinalis* |  |  |  |  |  |
| Snail density | 1 | 0.0004 | — | 2.69 | 0.10 |
| Pool (snail density) | 1 | — | 0.02 | 44.84 | < 0.0001 |
| Error | — | — | 0.01 | — | — |
|  |  |  |  |  |  |
| *Ulva* *lactuca* |  |  |  |  |  |
| Snail density | 1 | 0.0023 | — | 19.27 | < 0.0001 |
| Pool (snail density) | 1 | — | 0.06 | 57.33 | < 0.0001 |
| Error | — | — | 0.02 | — | — |
|  |  |  |  |  |  |
| *Fucus* spp. |  |  |  |  |  |
| Snail density | 1 | 3.05E-5 | — | 0.00 | 0.95 |
| Pool (snail density) | 1 | — | 0.07 | 76.68 | < 0.0001 |
| Error | — | — | 0.02 | — | — |
|  |  |  |  |  |  |
| *Spongomorpha* |  |  |  |  |  |
| Snail density | 1 | 3.21E-5 | — | 0.10 | 0.75 |
| Pool (snail density) | 1 | — | 0.02 | 4.49 | < 0.05 |
| Error | — | — | 0.01 | — | — |
|  |  |  |  |  |  |
| *Polysiphonia* |  |  |  |  |  |
| Snail density | 1 | 6.52E-5 | — | 0.08 | 0.78 |
| Pool (snail density) | 1 | — | 0.02 | 25.90 | < 0.0001 |
| Error | — | — | 0.01 | — | — |
|  |  |  |  |  |  |
| *Chondrus* |  |  |  |  |  |
| Snail density | 1 | 3.21E-05 | — | 0.10 | 0.75 |
| Pool (snail density) | 1 | — | 0.00 | 4.49 | 0.03 |
| Error | — | — | 0.01 | — | — |
